# Emerging roles of 3D-culture systems in tackling tumor drug resistance

**DOI:** 10.20517/cdr.2023.93

**Published:** 2023-11-21

**Authors:** Amin Nikdouz, Francesca Orso

**Affiliations:** Department of Translational Medicine, University of Eastern Piedmont, Novara 28100, Italy.

**Keywords:** Drug resistance, 3D culture system, extracellular matrix, tumor microenvironment, personalized medicine

## Abstract

Drug resistance that affects patients universally is a major challenge in cancer therapy. The development of drug resistance in cancer cells is a multifactor event, and its process involves numerous mechanisms that allow these cells to evade the effect of treatments. As a result, the need to understand the molecular mechanisms underlying cancer drug sensitivity is imperative. Traditional 2D cell culture systems have been utilized to study drug resistance, but they often fail to mimic the 3D milieu and the architecture of real tissues and cell-cell interactions. As a result of this, 3D cell culture systems are now considered a comprehensive model to study drug resistance *in vitro*. Cancer cells exhibit an *in vivo* behavior when grown in a three-dimensional environment and react to therapy more physiologically. In this review, we discuss the relevance of main 3D culture systems in the study of potential approaches to overcome drug resistance and in the identification of personalized drug targets with the aim of developing patient-specific treatment strategies that can be put in place when resistance emerges.

## INTRODUCTION

One of the most significant challenges in cancer treatment is drug resistance. Cancerous cells can possess an intrinsic resistance to therapy or they can develop an acquired resistance to chemotherapy and other targeted therapies, leading to treatment failure and disease progression^[1-3]^. The development of drug resistance in cancer cells is a multifactor event and involves numerous mechanisms that allow these cells to evade the effect of treatments. Intrinsic drug resistance can be defined as the innate resistance to drugs that are present inside the cells prior to the administration of any kind of treatment. It can be caused by (i) inherent genetic mutations that impair the response of cancer cells to drugs; (ii) intratumoral heterogeneity characterized by the presence of subclones, including cancer stem cells, which are insensitive to drugs; (iii) activation of defense mechanisms against environmental toxins. On the other hand, acquired resistance is defined as the progressive reduction of the efficacy of a treatment after its administration, which can be due to: (i) the surge of secondary mutations in proto-oncogenes that could become the new driver genes; (ii) mutation or dysregulation of the expression of the target of the therapy; (iii) dynamic alterations in the tumor microenvironment (TME). The mechanisms underlying intrinsic and acquired drug resistance could co-exist during tumor progression^[3-5]^. During the last decades, many different therapeutic approaches to overcome drug resistance have been tested. Combinatorial drug therapy (two or more drugs at the same time) has been used extensively and simultaneous multitargeting seems more effective in fighting drug resistance. It has been observed that cancers treated with the highest dosage of chemo- or targeted therapies rapidly become resistant to the treatment and new therapeutic approaches are based on “on and off” strategies. The intermittent treatment could interrupt the growth of resistant subpopulations inside the tumor^[6]^. Unfortunately, this kind of approach has also shown limitations and researchers are working hard to find new strategies to tackle drug resistance^[3]^. There is an urgent need to find new model systems to complement traditional 2D cell culture systems that are still the golden standard in the study of drug resistance. Researchers around the world are focusing on finding new systems to better model intrinsic and acquired resistance in tumors with the final aim of studying and tackling tumor drug resistance.

For years, cell culture systems have had an intense effect on the field of biomedical research for studying the molecular mechanisms of cancer progression and developing new therapies and treatments. Traditional 2D cell culture systems are the most commonly used preclinical models for different reasons: (i) easy to handle; (ii) relatively low costs; and (iii) suitable for high throughput analysis. Many anticancer drugs have been discovered thanks to the National Cancer Institute cancer cell line panel (NCI60), and the relevance of these models has been further supported by large pharmacogenomic screens such as the Genomics of Drug Sensitivity in Cancer, the Cancer Cell Line Encyclopedia, and the Cancer Therapeutics Response Portal^[7-10]^. Drug-adapted cancer cell lines are easily handled and they allow the establishment of a large number of models in a given timeframe and at a given cost. Even if the procedure is quite long (several months), the protocol is quite straightforward and the rate of success in establishing these models pretty high^[11]^. Clinically relevant mechanisms of resistance have been discovered using these models^[12]^; however, they cannot mimic intra-tumor heterogeneity, the 3D milieu, and the architecture of real tissues and cell-cell interactions. As a result, 3D cell culture systems are nowadays considered a more comprehensive model to study drug resistance *in vitro*. For example, the heterogeneous traits of a tumor, such as hypoxia, genetic status, and altered gene expression, can be more genuinely analyzed in 3D models rather than in 2D models^[13,14]^. Consequently, using 3D cell culture systems can find more reliable and accurate outcomes. However, the setting of protocols for 3D models is quite a time-consuming procedure and not successful for all kinds of tumors.

## ADVANTAGES OF 3D CELL CULTURE SYSTEMS

3D cell culture systems have recently emerged as a better option than conventional 2D cell culture systems for disease modeling, drug screening, and cancer research. In the section below, we want to briefly discuss the advantages of 3D cell culture over 2D cell culture.

3D cell culture systems mimic the *in vivo* microenvironment in comparison to 2D cell culture systems since they contain a higher degree of cellular organization, cell-cell interactions, and extracellular matrix (ECM) components. 3D cell cultures show a well-defined geometry, which could be directly related to function. Furthermore, inside these cultures, proliferating, non-proliferating, and necrotic cells are present, as in intact human tumors. In 3D cell systems, multicellular and multi-layered systems can be created and exploited to study the interactions between different cell types^[15-17]^. A wide variety of organoids, involving mini-brains, intestinal tissue, and liver tissue, can be generated and organoids made of various cell types can mimic the organization of an organ, making them ideal for drug screening and drug response^[18]^. The cellular heterogeneity obtained in 3D cultures models mass transport limitations typical of solid tumors. This allows for the designing of more precise models of disease, which can help develop effective treatments^[19]^. On the same line, 3D cell culture systems offer the potential for personalized medicine, enabling the production of patient-specific organoids for personalized drug screening and treatment^[20]^.

3D cell culture systems can potentially offer better predictive outcomes for patients’ drug responses^[21]^. They are more suitable for evaluating drug bioactivity since they simulate the impacts of treatments compared to 2D cell cultures more precisely^[22]^. Additionally, they are a more biologically applicable system for toxicology screening, as 3D systems provide more tissue-like structures and better simulate patients’ status, which can help predict the effects of toxins more accurately^[23]^. They are ideal, for example, for studying the influences of nanoparticles on cells, providing a more realistic test environment for nanotoxicity studies^[24]^.

Cell shape and environment are recognized as crucial in determining cell behavior and gene expression of a cell. In epithelial tumors, for example, polarity is essential, together with the formation of tight junctions and desmosomes linked to cell proliferation and tumorigenesis. In a 3D context, cells acquire a “normal-like” architecture and gene expression profile, while 2D monolayers on artificial support fail to maintain the original epithelial cell characteristics^[25]^. Cancer stem cells, which are crucial for treatment response, are strongly dependent on the niche for differentiation. Inducing stem cell differentiation and tissue-specific functionality is a challenging process in 2D cell culture systems, while 3D cell culture systems can be used for this purpose^[26,27]^. An important feature of 3D cell cultures is the remarkable control over the growth and differentiation of cells in complex tissue or organ-like structures that could be achieved, aiding researchers in simulating multifaceted disorder organization or physiological environments^[21]^.

3D cell culture systems are better tools for studying cell migration and invasion. Cells in 2D cell culture systems often move in a flat 2D environment, while cells in 3D cultures can exhibit more natural 3D migration and invasion, allowing for more precise modeling of cancer metastasis^[28]^. Moreover, 3D cell culture systems can be used to study the impacts of mechanical forces on cancer cell movement^[29]^. In theory, 3D cell culture systems can also be used to create substantial quantities of functional tissue, which may be used for transplantation or tissue engineering. This is specifically useful for applications such as skin grafts and bone regeneration^[30]^.

Even though 2D systems have been extensively used to study drug resistance due to their easy handling and relatively low costs, in the last few years, 3D culture systems have attracted the interest of researchers. Acquired resistance relies on different mechanisms, such as secondary mutations of proto-oncogenes or mutations or dysregulation of the expression of the target of the therapy and alterations in the TME^[3]^. 3D systems could help in the study of this phenomenon. For example, RNA editing levels are significantly correlated with drug sensitivity in cancer cell lines and can be heavily influenced by tumor environment^[31]^. By studying RNA editing in 3D cell culture models, researchers are able to investigate the relationship between RNA editing and drug sensitivity/resistance in a more physiologically relevant context.

Table 1 briefly summarizes the main differences between 2D and 3D culture systems.

**Table 1:** Comparison of the main features of 2D and 3D culture systems

**Aspect**	**2D cell culture**	**3D cell culture**
Cell arrangement	Cells grow in a monolayer on a flat surface	Organoids, hydrogels, and other three-dimensional structures, such as scaffolds, allow cells to grow in a more natural spatial environment.
Cell-cell interactions	Limited	Improved, allowing for direct interaction between cells, cell signaling, and sophisticated cellular actions.
Cell-matrix interactions	Limited	An ECM that mimics the *in vivo* microenvironment interacts with cells, managing cell adhesion, migration, and differentiation.
Phenotypic expression	Changed compared to *in vivo* conditions	Provides a more accurate representation of *in vivo* environments, including tissue-specific gene expression, protein synthesis, and cellular responses.
Cellular functions	Basic, absent of tissue-specific functions and complex cell-cell interactions	It is possible to accomplish more intricate and organotypic processes, such as cell polarization, differentiation, barrier function, and the creation of tissue-specific substances.
Spatial organization	Homogeneous distribution of cells	Cells are capable of spatial organization, resulting in multicellular structures, gradients, and tissue-like structures.
Drug response	Useful to study different drug sensitivities in comparison to *in vivo* conditions	Improved cellular responses and interactions across various cell types provide better predictive capability for drug screening, enabling assessment of medication efficacy and toxicity.
Disease modeling	Limited capability to recapitulate intricate diseases and tissue interactions	Enables the creation of disease models that are more accurate, making it easier to research disease progression, find new drugs, and practice personalized treatment.
High-throughput screening	Well-suited for high-throughput analyses and screening purposes	Due to 3D culture’s intricacy and additional experimental requirements, it is typically less suitable for high-throughput screening.

ECM: Extracellular matrix.

## DIFFERENT TYPES OF 3D CELL CULTURE SYSTEMS

**Figure 1:**
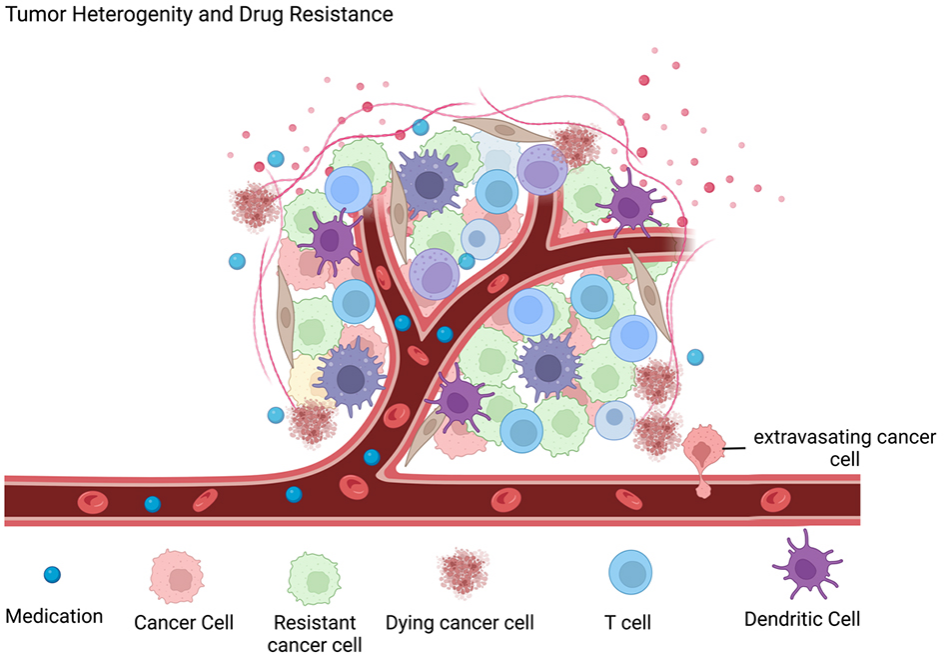
Tumor heterogeneity and drug resistance. Primary tumors are heterogeneous. Subpopulations of cancer cells showing partial or full resistance to therapy are present. The activation of resistance mechanisms can be due to the activation of genetic or epigenetic mechanisms that could be caused by the therapy itself and by the interaction of tumor cells with the microenvironment. This figure is generated with Biorender.com.

The most relevant components of the tumor stroma are ECM proteins and stroma cells. The ability of cancer cells to proliferate, migrate, adhere, differentiate and the activation of specific cell signaling pathways strongly rely on changes in ECM composition^[32]^ and on the interactions of tumor cells with stroma cells^[33]^. Cancer-associated fibroblasts (CAFs) are the most representative component in the tumor stroma, and they enhance cancer cell survival and the ability of cell invasion. They are involved in ECM remodeling and tumor metabolic rewiring, and contribute to the onset of drug resistance^[34]^. Mesenchymal stem cells (MSCs), which support the epithelial mesenchymal transition (EMT) of various cancer cells, alter the immunocompetence of the TME, inducing drug resistance in tumor cells. Various immune cells such as tumor-associated macrophages (TAMs), lymphocytes, natural killer (NK) cells, and dendritic cells (DCs) have pivotal roles in tumor control. Crosstalk between endothelial cells (ECs) and tumor cells during the formation of new blood vessels is crucial in providing the required nutrients and oxygen for the growth of tumors^[33]^ [Figure 1]. Considering all these important interactions within the TME, cell-based 3D models have emerged as models which could closely recapitulate physiological tumor organization *in vitro*.

**Figure 2:**
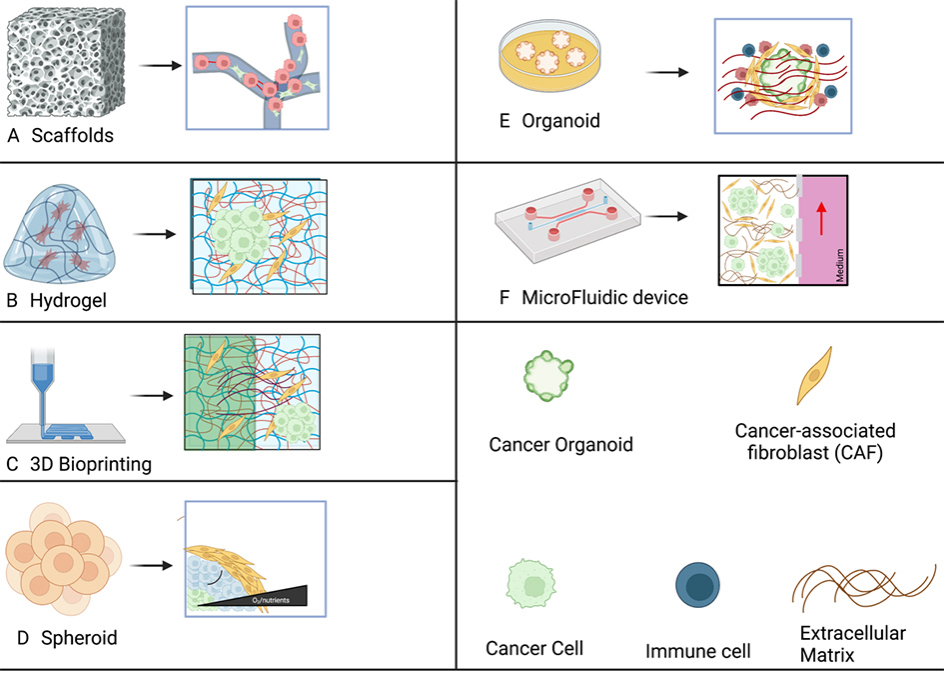
Schematic drawing of different 3D culture systems describing the main features of each system. (A) Scaffold. Cells, including immune cells, adhere to the scaffold, where they can proliferate and produce ECM, which coats the entire scaffold; (B) Hydrogel. Cells are combined with a hydrogel solution, which produces a sturdy framework that can support the cells. Cells divide and reorganize themselves; (C) 3D bioprinting. Cells are combined with hydrogel to produce bioink, a substance that can be printed using a 3D bioprinter. Depending on the type of bioink used, multiple cultures, including scaffold-based, scaffold-free, and semi-scaffold-free cultures, can be obtained; (D) Spheroid. Cells with significant cell-cell interaction are allowed to aggregate. As cells multiply, they rearrange themselves, and dense spheroids with oxygen or nutrition gradients could develop. Different kinds of cells can be combined in the spheroid; (E) Organoid. Cells are cultured in a hydrogel environment utilizing materials like Matrigel. Organoids are produced and are surrounded by other cells that replicate and produce natural ECM. Immune cell penetration can be simulated by adding immune cells to the culture; (F) Microfluidic device. Cells, spheroid, or organoids are plated onto the ready platform together with hydrogel, and then a medium containing nutrients can flow and perfuse the cells. Different kinds of cells can be added to the culture. This figure is generated with Biorender.com. ECM: Extracellular matrix.

Essential cell-ECM interactions can be recapitulated by biomimetic scaffolds, where tumor cells are seeded inside a 3D platform made of a porous biomaterial where they attach, start to grow, rearrange, and secrete ECM. After this process, called “scaffold maturation”, the entire scaffold is completely covered by cells [Figure 2A]. Biomaterials present in scaffolds can be synthetic polymers, i.e., polyethylene glycol (PEG), polycaprolactone (PCL), poly(hydroxyethylmethacrylate) (PHEMA), poly(lactic-co-glycolic acid) (PLGA), and ceramics (i.e., hydroxyapatite or bioglass), which are often preferred over natural polymers because their properties can be more easily controlled^[35]^ and they can be functionalized by the addition of peptides which can modulate protein adsorption as well as cell adhesion^[36]^. As far as natural biomaterials are concerned, collagen, fibrin, alginate, and chitosan can be derived from tissue and cells^[35]^. Decellularized native tissues potentially allow for an easier recapitulation of tumor tissue and ECM architecture being closer to the *in vivo* condition; however, the decellularization process can be challenging since various steps (i.e., detergent and enzyme digestion) may affect tissue architecture.

Hydrogels are crosslinked networks formed by hydrophilic polymers connected through physical, ionic, or covalent interactions that highly resemble ECM^[37]^ and allow cells to behave and communicate in an *in vivo* setting^[35]^. At first, cells are mixed with a precursor hydrogel, and then the hydrogel is crosslinked in order to obtain a cell-laden hydrogel. Cells start to grow and rearrange in the mature hydrogel, where they form cell clusters and secrete ECM, while the original hydrogel architecture remains intact [Figure 2B].

Scaffolds that are used to create complex 3D models can be obtained by 3D bioprinting, with the advantages of well-defined architecture, composition, and high reproducibility^[38]^. A cell-laden bioink made of a precursor hydrogel and cells is prepared and deposited in a preprogrammed pattern using a 3D bioprinter; hydrogel is then crosslinked to form the final structure [Figure 2C]. Printing may be obtained through extrusion, inkjet, and stereolithography, as well as laser-assisted and electrospinning-based bioprinting^[39]^. Scalability is one of the interesting features of 3D printed models. Indeed, it is possible to take into consideration 4D variables. Time, for example, is crucial for assessing the kinetics of growth factors, drugs or for following tumor cell dissemination over time^[40]^. Prominent issues in the use of 3D printing techniques could be damaging pressure and excessive heating during the printing of living cells, as well as slow printing speeds and the continuous need for new biocompatible and printable bioinks^[41]^. At the moment, however, insufficient reproducibility to create reference models is the main limitation. Despite these drawbacks, 3D bioprinting is a powerful technique since cancer and stroma cells are embedded in a complex microenvironment where tumor-stroma cell interactions, tumor-ECM contacts, and self-organization of the tissue could be deeply studied.

Spheroids [Figure 2D] contain important features that characterize tumors, such as cellular heterogeneity, gene expression variations, cell signaling pathway alterations, cell-cell, cell/ECM interactions, and multicellular layer organization, thus mimicking *in vivo* tumor morphology^[42]^. For these reasons, they are widely used to study tumor biology and to evaluate anticancer drugs. Spheroids can be generated easily by hanging drop methods or spontaneous cell aggregation of cells grown on low-attachment surfaces; however, more sophisticated techniques, such as 3D bioprinting or magnetic levitation, are applied to obtain more homogenous spheroid populations both in size and number^[43]^. Bigger spheroids (500 μm in diameter) can be used to recapitulate the milieu where micro-metastases develop since nutrients and oxygen are limited in those big structures^[44]^. Spheroids are useful models to study cancers that form tumor embolus or cancers characterized by packed tumor cell clusters such as inflammatory breast cancers^[45,46]^. In recent years, spheroid models have been implemented by the combination of different cell types in the same spheroids, i.e., tumor cells, monocytes, and CAFs, taking advantage of a particular technique utilizing spinner flasks^[47]^. In order to evaluate the role of pancreatic stellate cells (PSCs) in pancreatic ductal adenocarcinoma (PDAC) cells resistance to gemcitabine and c-MET inhibitors, 3D-spheroid co-cultures of primary human PDAC cells and PSC (heterospheroids) were generated and compared with spheroids containing only PDAC cells (homospheroids). While heterospheroids were more resistant to gemcitabine compared to homospheroids, no difference was observed for c-MET inhibitor treatment, suggesting that the choice of 2D or 3D systems to study drug resistance is strongly dependent on the kind of drug under investigation^[48]^. The main limitations of the use of spheroids are the poor uniformity in size/morphology of the obtained spheroids and often the difficulty in retrieving the cells for further molecular analysis^[46]^.

Spheroids are formed by forcing cell aggregation^[49]^, whereas organoids are obtained from progenitor cells that can proliferate, differentiate, and self-organize, thus closely recapitulating the 3D structure of the *in vivo* tissue/tumor from which they originate. As a result of this natural process, organoids preserve cancer cell heterogeneity as well as genetic and phenotypic properties of the tumor of origin [Figure 2E]. The significant feature of organoids is that they can be obtained from the patient’s cells and represent a potential alternative to patient-derived tumor xenografts (PDX) and animal models in many aspects, i.e., they are less laborious and expensive^[50]^. However, it has been observed that organoids could be helpful in predicting the responsiveness to therapy for some kinds of drugs but not for all. Ooft *et al.* developed patient-derived organoids (PDOs) from metastatic lesions of colorectal cancer (CRC) to identify non-responders to CRC standard therapy. While PDOs were able to predict the response in more than 80% of patients treated with irinotecan-based therapies, they failed to predict the response to 5-fluorouracil plus oxaliplatin, underlying once again the relevance of the choice of the system to study drug resistance^[51]^. To implement organoid models, co-culture of PSCs and PDAC tumor organoids has been established, thus allowing the differentiation of PSCs into myofibroblast-like and inflammatory CAFs^[52]^. More sophisticated organoid cultures can be obtained by 3D bioprinting^[53]^. The main drawback of organoids is that, in some kinds of tumors, they are unable to reach *in vivo*-like levels, and often, the variability can be high between experiments. Lastly, organoids lack vasculature and stroma^[18]^.

Both cell-ECM and cell-based 3D models described so far have an important limitation: cells cannot be perfused inside the 3D organization. Microfluidics devices have been introduced in tumor modeling to overcome this issue. They consist of a network of channels that allows the control and modification of various parameters, for instance, mechanical forces, cell localization, and chemical gradients [Figure 2F]. Spheroids or organoids can be grown inside these platforms and thanks to the control of several factors, microfluidics can model tumor tissues and organs so these systems are called organ-on-a-chip devices. Another important point is that a small volume of reagents can be used due to miniaturization, thus reducing the costs of these kinds of cultures compared to the other 3D-culture methods^[54]^. High-throughput screenings can be performed with increased controllability in microfluidics. The advent of 3D bioprinting has speeded up the process and reduced the costs. However, there is an urgent need for new materials to produce the chips, since the most common material used for this, polydimethylsiloxane (PMDS), can absorb small molecules in a nonspecific way. Microfluidics can be used to study tumor-stroma cell interactions because different cell types can be cultured in a chip^[54]^. The possibility to tightly control multiple gradient candidates makes microfluidics the model of choice to analyze the effects of growth factors or drugs in a biomimetic microenvironment. The main limitation of microfluidics is that the fabrication of these devices requires specialized skills.

Table 2 highlights the main characteristics of the different 3D systems described above.

**Table 2:** Comparison among the different 3D-culture techniques

**Technique**	**Description**	**Advantages**	**Disadvantages**	**Ref.**
Scaffolds	Cells are grown on or inside a three-dimensional scaffold structure	Mimics the structure of tissue and encourages cell differentiation	Limited control over the characteristics of the scaffold	[37]
3D bioprinting	A bioprinter is used to deposit cells layer by layer to produce intricate three-dimensional structures	Precise cell placement control; customizable	High-priced; limited scalability	[40]
Spheroids	Without an external scaffold, cells self-assemble into 3D spherical structures	Easy and inexpensive to produce; repeatable	Limited scalability, limited nutrient, and oxygen diffusion	[54]
Organoids	Three-dimensional self-organizing cell formations that resemble the functions and structures of organs	Reflects the complexity of the organ and models disease	Variability, complexity, and time-consuming generation	[18]
Microfluidics	Microscale channels that simulate *in vivo* tissue settings are used to cultivate cells, giving researchers great control over the culture environment.	High-throughput screening and accurate gradient control	Required technical knowledge; restricted scalability	[53]

## APPLICATIONS OF 3D CULTURE SYSTEMS IN DRUG RESISTANCE RESEARCH

3D cell culture systems have drawn substantial attention in the study of various aspects of drug resistance. Drug response and the mechanisms of drug resistance can be assessed using 3D cell culture models in a setting that is more physiologically suitable. Altered cell signaling, genetic variations, and tumor microenvironment interactions contribute to lower drug sensitivity due to tumor heterogeneity^[55,56]^.

3D cell culture platforms can be used for the screening and identification of new therapeutic agents that can overcome drug resistance. Through the use of these models, compound libraries may be tested in a more accurate tumor microenvironment, which helps researchers find new treatment combinations that are more effective at eliminating cancer cells that show intrinsic or acquired resistance. Ultimately, organoids and tumor explants are examples of patient-derived 3D cell culture models that can be used to assess how each patient responds to a particular treatment. With the use of this strategy, precision oncology techniques can be guided by the discovery of individualized treatment plans and the prediction of patient-specific drug responses^[55]^.

### 3D cell culture systems to overcome drug resistance

Intratumor heterogeneity poses a significant obstacle to effective cancer therapy. Tumor subpopulations may respond differently to therapeutic interventions due to intrinsic drug resistance or the emergence of acquired drug resistance. Therefore, innovative research models to comprehend and address this complexity are needed.

CRC exhibits distinct subpopulations within clonal organoids: cells generating large spheroids (D-pattern) and cells generating small spheroids (L-pattern). S-pattern spheroids display chemotherapy resistance, but modulation of Notch signaling can push them towards the D-pattern, offering a potential therapeutic target to reverse chemoresistance^[57]^. The nuclear tyrosine-protein kinase receptor 3 (TYRO3) receptor tyrosine kinase has been identified as an inducer of drug resistance and metastasis in CRC organoid culture and mouse models. TYRO3 function requires matrix metalloproteinase-2 (MMP-2) and bromodomain-containing protein 3 (BRD3), making selective inhibition of MMP-2 or BRD3 activity a potential strategy to ameliorate CRC malignancy^[58]^. In understanding drug resistance mechanisms, mutational status plays a crucial role. For instance, KRAS codon G12 (KRASG12) mutations in metastatic CRC (mCRC) patients have been associated with increased resistance to trifluridine/tipiracil chemotherapy. This observation was paralleled in isogenic cell lines and PDOs, highlighting the relevance of 3D systems in studying drug resistance^[59]^. In microsatellite instability-high (MSI-H) CRC, inflammation plays a pivotal role in disease progression and immunosuppression. *In silico* investigation highlighted a correlation between inflammatory conditions and poor response to programmed cell death-1 (PD-1) blockade. Cultures of paired T cells and organoid cells from patients confirmed this hypothesis, and single-cell RNA sequencing revealed the involvement of neutrophils in the suppressive immune microenvironment. An elevated neutrophil/lymphocyte ratio was associated with an impaired immune status and a poor response to immunotherapy, suggesting it could potentially serve as an indicator for clinical decision-making^[60]^. In MSI-H CRC, resistance to immune checkpoint blockade has been observed in a specific subtype characterized by peritoneal metastases and ascites formation. To study the mechanism of immune checkpoint blockade, PDOs were transplanted into the cecum of humanized mice. It was found that immune checkpoint blockade led to reduced tumor masses and liver metastasis, driven by the formation of tertiary lymphoid structures (TLS) containing B cells, T cells, and an activated interferon-γ signaling pathway. However, peritoneal metastases lacked B cells and TLS, and T cells displayed a dysfunctional phenotype^[61]^.

3D cell culture systems, such as organotypic tumor spheroids and matched PDOs, have been instrumental in identifying effective treatment strategies to overcome resistance to cancer immunotherapy in other cancers. For instance, Sun *et al.* identified TANK-binding kinase 1 (TBK1) as a potent therapeutic target to enhance the response to PD-1 blockade in melanoma and other cancers. Inhibition of TBK1 reduced the cytotoxicity threshold to effector cytokines, thereby empowering the response to PD-1 blockade^[62]^.

Resistance to common chemotherapy treatments, such as 5-fluorouracil and cisplatin (5FU + CDDP), remains a major challenge. As mentioned above, RNA editing is correlated with the emergence of resistance^[31]^; organoid lines from resistant patients with the intestinal subtype of gastric cancer (GC) showed upregulation of JAK/Src-signal transducer and activator of the transcription (STAT) signaling and adenosine deaminases acting on RNA 1 (ADAR1), along with hyper-edited lipid metabolism genes due to A-to-I editing on the 3’UTR of stearoyl- CoA desaturase (SCD1). SCD1 favored lipid droplet formation, reducing chemotherapy-induced ER stress and enhancing self-renewal in resistant GC lines^[63]^. In another subset of GC known as stem-like/Epithelial-to-mesenchymal transition/Mesenchymal (SEM-type) GC, resistance to glutaminolysis inhibition was observed due to a stem-like population in the tumor. SEM tumors displayed high glutaminase (GLS) levels and upregulation of the 3 phosphoglycerate dehydrogenase (PHGDH)-mediated mitochondrial folate cycle pathway to produce NADPH. A potential treatment strategy to combat chemotherapy resistance in SEM-type GC involves the combined inhibition of GLS and PHGDH to eliminate stemness-high cancer cells^[64]^.

Chemoresistance in PDAC is quite common, and there is an urgent need to identify new targets and compounds to improve treatment outcomes. A biobank of human PDAC organoid models was established and used to screen FDA-approved compounds, leading to the discovery of irbesartan, an angiotensin type 1 (AT1) receptor antagonist. Irbesartan inhibits the Hippo/YAP1 pathway, reducing c-Jun expression and enhancing chemotherapy effectiveness in killing PDAC cells. High c-Jun expression in PDAC patients was associated with poor response to the standard chemotherapy regimen (gemcitabine plus nab-paclitaxel)^[65]^. Loss of cyclin dependent kinase inhibitor 2A (CDKN2A) (encoding p16INK4A) and activation of KRAS play crucial roles in PDAC development and malignant growth. Restoration of p16INK4A with CDK4/6 inhibitors (CDK4/6i) alone has shown limited efficacy in clinics. However, combinatorial treatment with a CDK4/i and an ERK-MAPK inhibitor synergistically suppresses tumor growth through blocking CDK4/6i-induced compensatory upregulation of ERK, PI3K, antiapoptotic signaling, and MYC expression in PDAC cell lines and organoids^[66]^. Neoadjuvant chemotherapy (neoCTx) is used to treat PDAC, but its effects vary among patients. PDOs generated from PDAC tissues allowed researchers to evaluate differential responses to FOLFIRINOX or Gem/Pac regimens. This approach could help personalize poly-chemotherapy regimens, avoiding severe side effects and increasing the number of patients who benefit from complete neoadjuvant treatment^[67]^.

TME strongly influences the treatment outcome and new therapies targeting the cells of the TME are emerging. PDAC and cholangiocarcinoma (CCA) progression and chemoresistance are influenced by CAFs. In co-cultures of primary PDAC organoids and patient-matched CAFs, CAFs displayed a pro-inflammatory phenotype, while organoids showed increased expression of genes associated with EMT and drug resistance. This suggests that targeting CAFs could improve treatment sensitivity in PDAC and CCA^[68]^. CAFs also contribute to drug resistance in CCA. In PDOs consisting of epithelial and matched CAFs, CAFs were relatively resistant to bortezomib treatment due to an overexpression of CXCR4. However, the addition of a CXCR4 inhibitor reversed the resistance to bortezomib in CAFs and sensitized CCA to anti-PD1 treatment, offering a promising triple treatment strategy for CCA patients^[69]^.

Pyroptosis is a gasdermin-driven lytic programmed cell death triggered by inflammatory caspases that can be put to good use to kill cancer cells, including those exhibiting chemo- or targeted therapy resistance. Su *et al.* have demonstrated that pyroptosis can be reactivated in resistant pancreatic and lung cancer cell lines and organoids by administering a Src or ceramidase inhibitor. In resistant cancer cells, the β5-integrin protein plays a crucial role in controlling chemotherapy-induced pyroptosis, leading to chemoresistance. This effect is mediated through the upregulation of the sphingolipid metabolic enzyme ceramidase (ASAH2) expression, which is regulated by the STAT3 signaling pathway. The increased ceramidase expression results in a reduction of the metabolite ceramide concentration and subsequent suppression of reactive oxygen species (ROS) production, effectively blocking chemotherapy-induced canonical pyroptosis^[70]^.

In conclusion, intratumor heterogeneity presents a significant obstacle to the development of effective cancer therapies. There are now more ways to understand and deal with this complexity thanks to the use of sophisticated models such as organoids and 3D cultures. These models have highlighted possible treatment targets and drug combinations to potentially overcome drug resistance.

### 3D cell culture systems to investigate drug resistance mechanisms

Inhibitors of receptor tyrosine kinases (RTKIs) are commonly used in cancer treatment. However, in head and neck squamous cell carcinoma (HNSCC), despite the high expression of epidermal growth factor receptor (EGFR), RTKI treatment often fails to show therapeutic efficacy in clinical trials, questioning their inclusion in standard therapy regimens. To understand the reasons behind these failures, researchers evaluated the response of HNSCC cell lines to RTK inhibitors under both 2D and 3D cell culture conditions. Interestingly, the HNSCC cells displayed strong resistance to lapatinib, an RTKI, when cultured in 3D conditions. However, in a 2D setting, the same cells responded to the lapatinib treatment. This resistance was associated with an overexpression of HER3. These results indicate that the increased cell-to-cell contacts and enhanced communication between cells due to higher cell density, as well as the augmented concentration of receptors and intracellular signaling molecules of the EGFR family in 3D systems, could impact drug response. Moreover, in 3D systems, cells live in hypoxic and nutrient-poor conditions and behave as dormant cells that are less susceptible to cytostatic treatment, leading to increased resistance. This finding indicates that the culture conditions can alter cell signaling pathways, potentially leading to different drug resistance mechanisms in cancer therapy^[71]^.

Glioblastoma (GBM) is a challenging cancer with poor patient prognosis and frequent tumor recurrence. Due to the resistance of certain subpopulations, such as mesenchymal and glioma stem cells, to the standard chemotherapy drug temozolomide (TMZ). Small protein kinase inhibitors have also been extensively studied for GBM treatment, but their benefit for patients has been limited compared to standard therapy regimens. Fabro *et al.* investigated the effects of prolonged treatment with TMZ, enzastaurin, and imatinib on patient-derived GBM 2D and 3D organotypic multicellular spheroid models. They observed a heterogeneous inter-patient response to the different drugs, with minor changes in kinase activation, primarily associated with the ErbB signaling pathway. Additionally, they identified a new resistance mechanism to imatinib treatment in one 3D sample, resulting in a more invasive behavior. The authors suggest the stroma cell interactions present in 3D structures could exert a protective effect on tumor cells against TMZ action^[72]^. In other studies, using 3D collagen scaffold culture and 3D Ca-alginate scaffolds, researchers analyzed the gene expression profiles of GBM cells. Glioma cells cultured in 3D collagen scaffolds exhibited increased colony and sphere formation and increased drug resistance compared with 2D cultures. The hub genes involved in 3D collagen-induced drug resistance (AKT1, ATM, CASP3, CCND1, EGFR, PARP1, and TP53) were predicted by bioinformatics and their expression was verified by Western Blot analysis^[73]^.

Glioma cells cultured in 3D Ca-alginate scaffolds exhibited significant changes in gene expression compared with 2D cultures, with upregulation of genes related to mitogen-activated protein kinase signaling, autophagy, drug metabolism through cytochrome P450, and ATP-binding cassette transporter, and downregulation of genes related to the cell cycle and DNA replication. This altered gene expression could be due to the fact that the 3D-collagen culture may enhance the stemness traits of glioma cells compared to 2D conditions^[74]^. These findings provide valuable insights into the differences in gene expression between 2D and 3D culture systems and their potential implications for drug resistance in GBM.

Biomimetic collagen scaffolds have been used as models to study the tumor hypoxic state and may be valuable tools in predicting chemotherapy responses in breast cancer (BC). Triple negative (TN) and luminal A BC cells were treated with doxorubicin in 2D cultures, 3D collagen scaffolds, or orthotopically transplanted murine models. In 3D culture conditions, TN cells displayed impaired drug uptake, increased drug efflux, and drug lysosomal confinement, contributing to drug resistance. Luminal A cells, on the other hand, were found to be insensitive to DNA damage due to deregulation of the p53 pathway. Transcriptome analysis identified a signature of deregulated genes that were validated in BC patients, showing their potential as predictive biomarkers. Transporter associated with antigen processing 1 (TAP1) and tumor protein p53-inducible protein 3 (TP53I3) showed high expression and were associated with shorter relapse in ER+ breast tumor patients, while high expression of lysosomal-associated membrane protein 1 (LAMP1) was associated with the same clinical outcome in TNBC patients. Resveratrol treatment with subsequent hypoxia inhibition partially re-sensitized cells to doxorubicin treatment, highlighting the relevance of these 3D preclinical models in the study of resistance mechanisms. Conversely, data obtained in monolayers were not able to recapitulate *in vivo* conditions and efficacy was overestimated when tested in a 2D context^[75]^. To investigate recurrence mechanisms in ER+ BC, tumor organoids were generated from needle biopsy, surgical excision, and cerebrospinal fluid samples. Next generation sequencing (NGS) analysis revealed detrimental mutations in PIK3CA and TP53 genes and mutations of unknown functions in BAP1, RET, AXIN2, and PPP2R2A. Drug screening in BC organoids allowed for the evaluation of drug toxicity and showed dynamic changes in tumor progression, reflecting the heterogeneity of BC and demonstrating their reliability as models for personalized medicine development^[76]^. In ER+ positive BC cells, the recurrent deletion of 16q12.2 affects AKTIP, which governs tumorigenesis, specifically in ER+ positive BC cells. Its deletion is linked to ERα protein level and activity and triggers JAK2-STAT3 activation, an alternative survival signal in the absence of ERα activation. Consequently, ERα-positive PDOs become more resistant to ERα antagonists, but this resistance can be overcome by co-inhibition of the JAK2/STAT3 signaling pathway^[77]^.

These papers show the applications of 3D cell culture platforms in the study of the mechanisms behind treatment resistance in various cancers in comparison with 2D systems. In order to design targeted therapies, it is now possible to identify specific molecular pathways and cellular interactions that contribute to resistance through the use of 3D models.

### Potential use of 3D cell culture systems to identify individual drug response

In recent years, personalized medicine has made considerable progress, allowing tailored treatments for each patient. One of the potential advancements in personalized medicine is the development of 3D cell culture models, which could hold the potential for addressing individual drug resistance.

Anderle *et al.* pioneered the creation of a 3D system of epithelial ovarian cancer (EOC) comprising patient-derived microtumors and autologous tumor-infiltrating lymphocytes (TILs). By employing reverse-phase protein array (RPPA) analysis of over 110 total and phospho-proteins on these models, they measured patient-specific sensitivities to standard platinum-based therapy and predicted individual treatment responses. The inclusion of autologous TILs in 3D cultures facilitated testing patients’ responses to immune checkpoint inhibitors (ICIs). The therapeutic sensitivity predictions obtained through 3D systems hold clinical relevance post-surgery for patients’ treatment. Ongoing follow-up studies in larger cohorts aim to validate the effectiveness of this platform for guiding clinical decision-making^[78]^.

A significant milestone was achieved by Senkowski *et al.*, who leveraged viably biobanked tissues to establish organoids from high-grade serous ovarian cancer (HGSC). These organoids faithfully recapitulated the original tumors in terms of both genetics and phenotype, as evidenced by genomic, histologic, and single-cell transcriptomic analyses. Furthermore, when cultured in a human plasma-like medium, organoid drug responses correlated with clinical treatment outcomes, highlighting the potential of these models for predicting patient responses to therapy^[79]^.

The significance of 3D systems in predicting drug responses and the development of resistance has led to the establishment of international consortia conducting multicenter studies to validate the clinical relevance of these models.

The INFORM program, an international precision oncology initiative, enrolled 132 pediatric cancer patients with relapsed or refractory conditions. In a two-year pilot study, fresh tumor tissue spheroid cultures were exposed to a library of clinically relevant drugs. The drug sensitivity profile (DSP) results from the multicellular tumor tissue spheroid cultures correlated with known molecular targets (BRAF, ALK, MET, and TP53 status). Remarkably, drug vulnerabilities were identified in 80% of cases, and the correlation between clinical outcomes and DSP results in selected patients suggests the potential advantage of this platform in predicting individual treatment responses^[80]^.

3D models are also proving useful in predicting individual responses to radiotherapy (RT) in various tumor types. For instance, Lee *et al.* cultured HNSCC patient tumor cells in a 3D pillar/well array culture system, exposing them to standard radiation protocols and evaluating their RT response. This approach allowed the quantification of the radioresponse index in HNSCC patients^[81]^. Similarly, a HNSCC organoid biobank comprising 110 models, including 65 tumor models, was established. Organoids exposed to chemo-radiotherapy and targeted therapies demonstrated drug response correlations with patient clinical outcomes. Notably, *in vitro*, organoid response to RT closely mirrored patients’ clinical responses^[82]^.

In the context of mCRC, which often develops resistance and has limited therapeutic options, tumor-derived organoids are being used to assess individual drug sensitivity and explore new treatment avenues. In a phase 2 study involving 90 mCRC patients with progression after standard therapy, organoids derived from metastatic biopsies were cultured and evaluated for sensitivity to a panel of drugs. Patients were treated with the drug demonstrating the highest relative activity, resulting in a 50% progression-free rate at two months^[83]^. Another study generated organoids from 40 mCRC patients and performed drug screenings, associating the results with patients’ responses. In the future, these findings may support the potential use of organoids to generate functional data and to aid in clinical decision-making^[84]^. The integration of PDO drug response with multi-omics data could likely lead to the identification of proteomic and gene expression signatures capable of predicting treatment response or resistance in advanced CRC. Drug sensitivity tests coupled with mass spectrometry and RNA-seq analysis revealed differential responses to oxaliplatin and palbociclib. Oxaliplatin resistance was linked to t-RNA aminoacylation processes, while high palbociclib responses were associated with MYC activation and T-complex protein ring complex (TriC) chaperonin protein enrichment^[85]^.

The complexity of protocols to generate PDOs has posed a challenge, prompting efforts to standardize these procedures. A novel microfluidics-based system known as the Pu·MA System has been introduced, coupled with high-content imaging and metabolite analysis, for the processing and multi-functional profiling of tumoroid samples from metaplastic BC subtype patients. High-content imaging and multi-parametric profiling revealed tumoroid sensitivity to specific drugs, closely mirroring primary tumor responses^[86]^. In inflammatory BC, a living organoid biobank was established from locally advanced patients undergoing neoadjuvant chemotherapy. Organoids treated with neoadjuvant drugs demonstrated a response pattern that closely matched patients’ clinical responses, suggesting that PDOs could predict neoadjuvant therapy outcomes in BC patients^[87]^.

Predicting clinical treatment responses in locally advanced or metastatic lung cancer (LC) patients using tumor organoids has also been explored. Wang *et al.* generated 212 LC organoids and conducted drug sensitivity tests for chemotherapy and targeted therapy. Organoids successfully predicted clinical treatment responses^[88]^. In another study by Mazzocchi *et al.*, 3D LC organoids were fabricated from pleural effusion aspirate, a rare cell source. These organoids recapitulated lung tissue anatomy and exhibited lung-specific behavior compared to 2D cultures. While 2D cultures were more sensitive to chemotherapy, organoids better reflected the *in vivo* situation, underlining the relevance of 3D systems in studying drug responses and resistance emergence^[89]^.

In the context of GBM, the need for assays that predict drug responses prompted the establishment of a high-density 3D primary cell culture model from resected GBM tissue. These cultures accurately modeled GBM heterogeneity, including tumor and surrounding cells, and replicated histopathological traits of parent tumors. These 3D cultures effectively predicted chemotherapy responses within a brief period and correlated with patients’ responses to TMZ therapy^[90]^. Metabolic imaging based on NAD(P)H fluorescence lifetime imaging microscopy (FLIM) was applied to GBM organoids to predict anticancer treatment responses. This technique identified TMZ Responder and Non-Responder tumors shortly after surgery, with metabolic stratification aligning with molecular levels, demonstrating its potential for patient stratification^[91]^.

PDOs have also proven valuable in predicting responses to neoadjuvant chemotherapy (NAT) in PDAC patients. A PDO biobank was generated from patients receiving NAT and untreated patients. The response to NAT correlated with PDO chemotherapy response (oxaliplatin), highlighting the feasibility of rapid PDO drug screening shortly after tissue resection for optimal patient NAT regimen selection^[92]^.

Through standardization and integration with multi-omics data, 3D cell culture models offer improved patient outcomes across various cancer types, enabling precise prediction of drug reactions, therapeutic outcomes, and personalized treatment options.

## CONCLUSION

Overall, 3D cell culture models may be of help in understanding the general and the patients’ specific mechanisms of drug resistance by providing more physiologically relevant systems for disease modeling and drug screening. These models could allow for the identification of personalized drug targets and the potential development of patient-specific treatment strategies. However, standardization of culture protocols, characterization methods, and outcome metrics is essential for maximizing the clinical value of 3D cell culture models. For example, the choice of bio-materials used to generate 3D cultures is critical for the successful generation of organoids and the prediction of drug response. The comparison among patient-derived BC cells encapsulated in bioprinted PEG-derived hydrogels and gelatin-derived hydrogels (GelMA and GelSH) showed that GelSH increased organoid formation ability and a better response to doxorubicin, EP31670, and paclitaxel treatments compared to 2D cultures and other matrices^[36]^. To ensure repeatability among laboratories, efforts are being made to create standards and quality control procedures^[93]^. Integrating 3D cell culture models with multi-omics data (genomics, transcriptomics, and proteomics) can provide a thorough understanding of disease causes and treatment responses. The combination of these datasets can enable the identification of molecular signatures and biomarkers for patient stratification and therapy choice^[94]^. However, the fact that the 3D models cannot be used for all drugs without distinction needs to be considered. It has been shown, for instance, that organoids fail to predict the response to 5-fluorouracil plus oxaliplatin, while they are able to predict the response in more than 80% of patients treated with irinotecan-based therapies^[51]^. Therefore, for some kinds of drugs, 2D culture models remain the elective systems to study the mechanisms of drug resistance^[12]^. Despite this, 3D cell culture models hold tremendous potential for improving therapeutic approaches and ultimately enhancing patients’ outcomes.

## DECLARATIONS

### Copyright

© The Author(s) 2023.
